# High Resolution Human Leukocyte Antigen Class I Allele Frequencies and HIV-1 Infection Associations in Chinese Han and Uyghur Cohorts

**DOI:** 10.1371/journal.pone.0050656

**Published:** 2012-12-12

**Authors:** Yanhou Liu, Zhongfang Zhao, Tianyi Li, Qi Liao, Nicholas Kushner, Neal Y. Touzjian, Yiming Shao, Yongtao Sun, Amie J. Strong, Yichen Lu

**Affiliations:** 1 Vaccine Laboratory, Nankai University, Tianjin, China; 2 Vaccine Technologies Inc., Wellesley, Massachusetts, United States of America; 3 National Center for AIDS Prevention and Control, Beijing, China; 4 Fourth Military Medical University, Tangdu Hospital, Xi'an, China; 5 Department of Immunology and Infectious Diseases, Harvard School of Public Health, Boston, Massachusetts, United States of America; University of Hawaii Manoa, United States of America

## Abstract

**Background:**

Host immunogenetic factors such as HLA class I polymorphism are important to HIV-1 infection risk and AIDS progression. Previous studies using high-resolution HLA class I profile data of Chinese populations appeared insufficient to provide information for HIV-1 vaccine development and clinical trial design. Here we reported HLA class I association with HIV-1 susceptibility in a Chinese Han and a Chinese Uyghur cohort.

**Methodology/Principal Findings:**

Our cohort included 327 Han and 161 Uyghur ethnic individuals. Each cohort included HIV-1 seropositive and HIV-1 seronegative subjects. Four-digit HLA class I typing was performed by sequencing-based typing and high-resolution PCR-sequence specific primer. We compared the HLA class I allele and inferred haplotype frequencies between HIV-1 seropositive and seronegative groups. A neighbor-joining tree between our cohorts and other populations was constructed based on allele frequencies of HLA-A and HLA-B loci. We identified 58 HLA-A, 75 HLA-B, and 32 HLA-Cw distinct alleles from our cohort and no novel alleles. The frequency of HLA-B*5201 and A*0301 was significantly higher in the Han HIV-1 negative group. The frequency of HLA-B*5101 was significantly higher in the Uyghur HIV-1 negative group. We observed statistically significant increases in expectation-maximization (EM) algorithm predicted haplotype frequencies of HLA-A*0201-B*5101 in the Uyghur HIV-1 negative group, and of Cw*0304-B*4001 in the Han HIV-1 negative group. The B62s supertype frequency was found to be significantly higher in the Han HIV-1 negative group than in the Han HIV-1 positive group.

**Conclusions:**

At the four-digit level, several HLA class I alleles and haplotypes were associated with lower HIV-1 susceptibility. Homogeneity of HLA class I and Bw4/Bw6 heterozygosity were not associated with HIV-1 susceptibility in our cohort. These observations contribute to the Chinese HLA database and could prove useful in the development of HIV-1 vaccine candidates.

## Introduction

Since the first case of AIDS was identified in 1981, over 21 million people have died from HIV infection. According to an estimate issued jointly by China's Ministry of Health and the Joint United Nations Program on HIV/AIDS (UNAIDS), by the end of 2011, there were an estimated 780,000 people living with HIV in China, including 154,000 with severe symptomatic AIDS [1]. The high prevalence of HIV and limited access to treatment for many in the developing world strongly underlines the need for a preventive or therapeutic HIV vaccine. In the design and development of an effective vaccine, one must consider the role of T cell immunity in HIV-1 infection. Over the past few years, a number of studies have demonstrated that CD8^+^ cytotoxic T lymphocyte (CTL) response plays a central role in immune control of HIV. The protective effects of CD8^+^ CTL responses in primary HIV-1 infections [2], [3] and in long term nonprogressors [4], [5] have been documented. Associations between HIV specific CD8^+^ CTL responses, and HIV exposed but uninfected individuals, were also observed [6], [7], [8]. Intracellular epitopes presented to CD8^+^ cells are usually bound by HLA class I (HLA-A, B, and Cw) molecules, which are encoded by HLA-I genes. The HLA genes comprise the most polymorphic loci in the human genome. At the population level, HLA diversity has an impact on susceptibility and the outcome of HIV-1 infection. This is based on associated diversity of antigen recognition and presentation [9], [10], [11], [12]. The relationship between the frequency of HLA genes within different racial and ethnic groups and HIV-1 susceptibility and disease progression deserves further investigation.

Recently, large investigations employing high resolution HLA typing of various Chinese populations have been conducted [13], [14], [15], [16], [17], [18], [19]. However, most of these studies focused exclusively on the distribution of HLA alleles and haplotypes, and not on their association with HIV infection. HIV infection and HLA profile association studies have been conducted in Chinese populations, but have been based on 2-digit HLA typing [20], [21]. The scarcity of research on the association between high resolution HLA alleles and haplotypes and HIV-1 susceptibility may be an impediment to the development of an effective HIV vaccine in China.

In this study, we carried out high resolution HLA class I typing on a Chinese Han cohort and a Chinese Uyghur cohort, most of whom were HIV-1 positive. Associations between HIV-1 susceptibility and host immunogenetics were investigated by analysis of allele frequencies and EM algorithm predicted haplotype frequencies in HIV-1 positive and HIV-1 negative groups. The allele frequencies were also compared to other Chinese populations. The purpose of this study was to contribute to the database of Chinese HLA allele and haplotype distribution, which may be useful in HIV vaccine development and in the selection of subject populations in future clinical trials.

## Results

### Allele distribution of HLA class I

From the 488 Chinese individuals, 58 HLA-A, 75 HLA-B, and 32 HLA-Cw distinct alleles were identified. In our Chinese Uyghur cohort, 21 common HLA-A alleles with frequencies higher than 0.01 accounted for 91.9% of the total HLA-A alleles. In addition, 28 HLA-B and 19 HLA-Cw alleles with frequencies higher than 0.01 comprised 88.5% of HLA-B and 95.3% of HLA-Cw alleles. In our Chinese Han cohort, 15 common HLA-A alleles with frequencies higher than 0.01 accounted for 93.0% of the total HLA-A alleles. Similarly, 26 HLA-B and 15 HLA-Cw alleles comprised 88.4% of HLA-B and 93.6% of HLA-Cw alleles, respectively (Table 1). Complete data are available at allelefrequencies.net. No novel alleles were identified in these two cohorts.

**Table 1:** Common HLA class I alleles in Chinese Uyghur and Han populations.

HLA-A			HLA-Cw			HLA-B		
	Frequency			Frequency			Frequency	
Allele	Uyghur	Han	Allele	Uyghur	Han	Allele	Uyghur	Han
0101	0.093	0.029	0102	0.053	0.170	0702	0.025	0.018
0201	0.134	0.130	0202	0.009	0.012	0705		0.015
0203	0.009	0.038	0302	0.019	0.072	0801	0.053	0.011
0205	0.031	0.003	0303	0.022	0.067	1301	0.012	0.038
0206	0.025	0.058	0304	0.065	0.075	1302	0.050	0.086
0207	0.031	0.073	0401	0.118	0.057	1402	0.037	0.003
0211	0.019	0.003	0501	0.019	0.005	1501	0.025	0.038
0301	0.096	0.031	0602	0.177	0.119	1502	0.009	0.029
1101	0.099	0.157	0701	0.043	0.006	1511	0.003	0.023
1102	0.012	0.015	0702	0.099	0.135	1525	0.003	0.012
2301	0.022	0.005	0801	0.025	0.087	1801	0.028	0.005
2402	0.115	0.136	0802	0.034	0.005	2705	0.006	0.012
2601	0.050	0.024	0803	0.006	0.011	3501	0.037	0.041
2901		0.017	1202	0.056	0.028	3502	0.022	0.005
3001	0.031	0.083	1203	0.078	0.009	3503	0.056	0.006
3004	0.012	0.002	1402	0.022	0.041	3701	0.019	0.012
3101	0.019	0.038	1403		0.011	3801	0.025	0.003
3201	0.019	0.014	1502	0.037	0.037	3802	0.019	0.038
3301	0.019	0.002	1505		0.015	3901	0.019	0.014
3303	0.034	0.086	1602	0.019		4001	0.031	0.069
6801	0.025	0.002	1701	0.019		4002	0.009	0.023
6802	0.016	0.002				4006	0.028	0.031
						4101	0.028	
						4402	0.022	0.005
						4403	0.043	0.026
						4601	0.025	0.107
						4801	0.016	0.031
						4901	0.012	
						5001	0.090	0.009
						5101	0.056	0.055
						5201	0.047	0.040
						5501	0.019	
						5502	0.006	0.014
						5701	0.025	0.011
						5801	0.019	0.067

Uygur 2n = 322. Han 2n = 654. Alleles with frequencies lower than 0.01 in both Uyghur and Han cohorts are not shown.

On the HLA-A locus of our Uyghur cohort, the HLA-A*02 group represented 27.3% of the total HLA-A alleles, followed by the A*24 group (12.4%) and the A*11 group (11.8%). In our Han cohort, the most predominant group was also the A*02 group (31.5%), followed by A*11 (18.7%) and A*24 (14.4%).

In total, 15 (15/161, 9.3%) Uyghur subjects were homozygous on HLA-A locus at 4-digit level high resolution typing. The four most frequent homozygous alleles in our Uyghur cohort were A*2402 (2n = 8), A*0201 (2n = 6), A*0101 (2n = 4) and A*1101 (2n = 4). Likewise, 46 (46/327, 14.1%) Han subjects were homozygous on the HLA-A locus at 4-digit level, including the three most frequent alleles A*1101 (2n = 26), A*2402 (2n = 24) and A*0201 (2n = 14).

The HLA-B locus was the most diverse among the three HLA class I loci. In our Uyghur cohort, HLA-B*35 (12.7%), B*50 (9.0%) and B*40 (7.1%) were the three most frequent groups on the HLA-B locus. In our Han cohort, HLA-B*40 (12.4%), B*13 (12.4%) and B*15 (12.1%) were the three most frequent groups. In our Uyghur cohort there were 8 (8/161, 5.0%) subjects homozygous on HLA-B locus at 4-digit level, and the most prevalent homozygous allele was B*5001 (2n = 4). In our Han cohort there were 27 (27/327, 8.3%) subjects homozygous on the HLA-B locus at 4-digit level, and the most prevalent homozygous allele was B*4601 (2n = 24).

On the HLA-Cw locus of our Uyghur cohort, the three most common groups were HLA-Cw*07 (20.2%), Cw*06 (17.7%), and Cw*12 (13.4%). The three most common groups on the HLA-Cw locus of our Han cohort were HLA-Cw*03 (21.4%), Cw*01 (17.6%) and Cw*07 (16.4%). At the 4-digit level, among 18 (18/161, 11.2%) HLA-Cw homozygous subjects in our Uyghur cohort, Cw*0602 (2n = 14) was the most common allele. Among the 40 (40/327, 12.2%) HLA-Cw homozygous subjects in our Han cohort, Cw*0102 (2n = 28) and Cw*0702 (2n = 20) were the predominant alleles. Altogether, 18 Han and 3 Uyghur individuals were homozygous on two loci, HLA-A-Cw, A-B, or Cw-B. Only 7 Han and 3 Uyghur individuals were homozygous on three loci, HLA-A-Cw-B.

### Haplotypes of HLA class I

The EM algorithm predicted HLA class I haplotypes with estimated frequencies higher than or equal to 2.0% are summarized in Table 2. In our Han cohort, two HLA-A-B haplotypes, three HLA-A-Cw haplotypes, and three HLA-Cw-B haplotypes had an estimated frequency greater than 5.0%. There were two HLA-A-Cw-B haplotypes with an estimated frequency higher than 5.0%. In our Uyghur cohort, only 2 HLA-Cw-B haplotypes had an estimated frequency higher than or equal to 5.0%, and no HLA-A-B, HLA-A-Cw, or HLA-A-Cw-B haplotype had frequencies higher than 5.0%.

**Table 2:** Common HLA class I haplotypes in Chinese Uyghur and Han populations.

	Han		Uyghur	
	Haplotype	Frequency	Haplotype	Frequency
HLA-A*-B*	3001 1302	0.070	0205 5001	0.028
	0207 4601	0.063	0201 5101	0.026
	3303 5801	0.049	3001 1302	0.022
	1101 4601	0.021	0301 5001	0.021
			0201 4403	0.021
HLA-A*-Cw*	3001 0602	0.072	2402 0304	0.034
	0207 0102	0.060	0301 0401	0.031
	3303 0302	0.053	0201 0602	0.028
	1101 0702	0.041	0205 0602	0.028
	1101 0102	0.041	1101 0401	0.023
	0201 0303	0.033	3001 0602	0.021
	2402 0304	0.026	2601 1203	0.021
	2402 0801	0.024	0101 0602	0.020
	0201 0801	0.022		
	0203 0702	0.021		
	0101 0602	0.020		
HLA-Cw*-B*	0102 4601	0.101	0602 5001	0.081
	0602 1302	0.086	0602 1302	0.050
	0302 5801	0.066	0702 0801	0.043
	0702 4001	0.036	1202 5201	0.043
	1402 5101	0.035	0802 1402	0.034
	0304 1301	0.034	0401 3503	0.031
	0702 3802	0.034	0401 3501	0.031
	0801 1502	0.029	0602 5701	0.025
	0304 4001	0.025	1203 3503	0.025
	0801 4006	0.024	0401 3502	0.022
	1202 5201	0.024	0702 0702	0.022
	0102 5401	0.021	0706 4403	0.022
	0303 1511	0.021	1203 3801	0.022
			1402 5101	0.022
HLA-A*-Cw*-B*	3001 0602 1302	0.070	0205 0602 5001	0.028
	0207 0102 4601	0.059	3001 0602 1302	0.022
	3303 0302 5801	0.049		
	1101 0102 4601	0.020		

Only haplotypes with estimated frequencies ≥0.02 are shown.

### HLA class I allele frequency comparison between HIV-1 positive and negative groups

**Figure 1:**
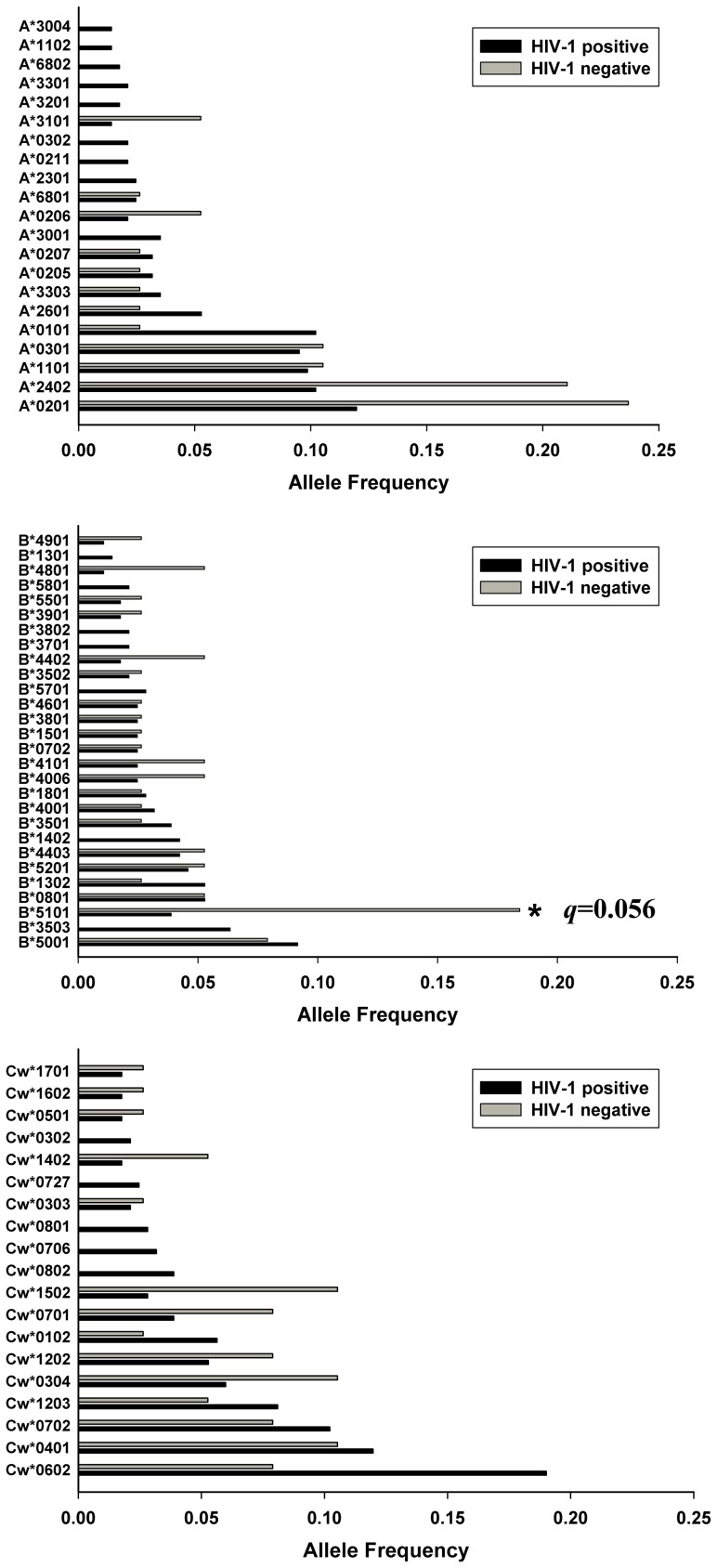
Common HLA class I allele frequencies comparison in Uyghur HIV-1 positive and negative cohorts. Only alleles with frequencies ≥0.01 are shown. The *q* values refer to comparisons between HIV-1 positive and HIV-1 negative groups.

**Figure 2:**
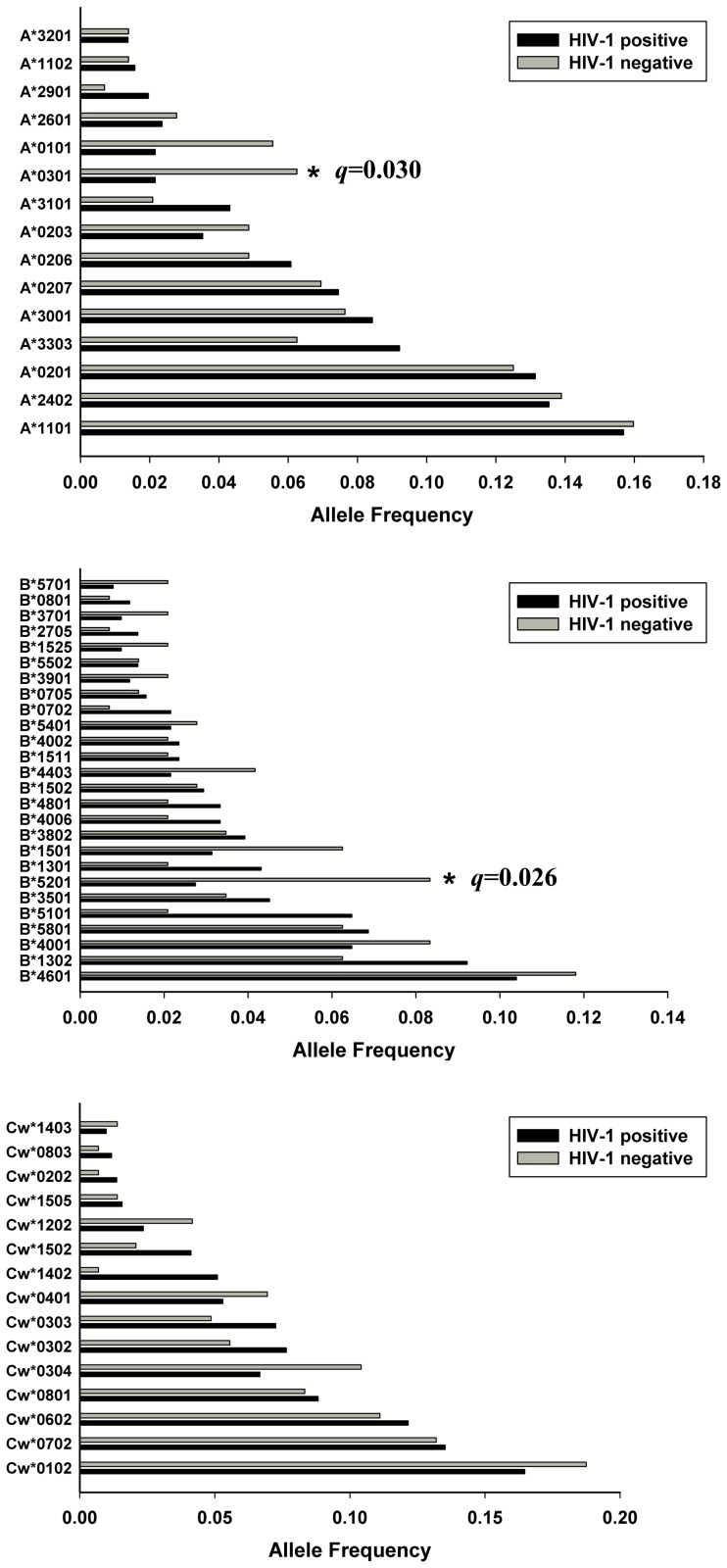
Common HLA class I allele frequencies comparison in Han HIV-1 positive and negative cohorts. Only alleles with frequencies ≥0.01 are shown. The *q* values refer to comparisons between HIV-1 positive and HIV-1 negative groups.

In our Uyghur cohort, the frequency of HLA-B*5101 (OR = 0.18, 95% CI: 0.06 to 0.49; *p* = 0.002, *q* = 0.056) was significantly higher in the HIV-1 negative group. We did not find HLA-A or HLA-Cw alleles with significantly different frequencies between the HIV-1 positive and HIV-1 negative groups (Figure 1). In our Han cohort, a statistically significant increase in allele A*0301 (OR = 0.25, 95% CI: 0.09 to 0.64; *p* = 0.002, *q* = 0.030) and B*5201 (OR = 0.24, 95% CI: 0.10 to 0.57; *p* = 0.001, *q* = 0.026) was observed in the HIV-1 negative group. We did not find significant differences in Han HLA-Cw allele frequencies when comparing the HIV-1 positive to the HIV-1 negative group (Figure 2). In the Uyghur cohort, among all common alleles with a frequency higher than 0.01, twenty-one alleles were found exclusively in the HIV-1 positive group (Table S1). In both of the Uyghur and Han cohorts, there were several low frequency alleles (frequency less than 0.01) found only in the HIV-1 positive group or HIV-1 negative group. None of the common alleles nor any of the low frequency alleles showed significant differences when comparing the HIV-1 positive group to the HIV-1 negative group (Tables S1 and S2).

### HLA class I haplotype frequency comparison between HIV-1 positive and negative groups

**Figure 3:**
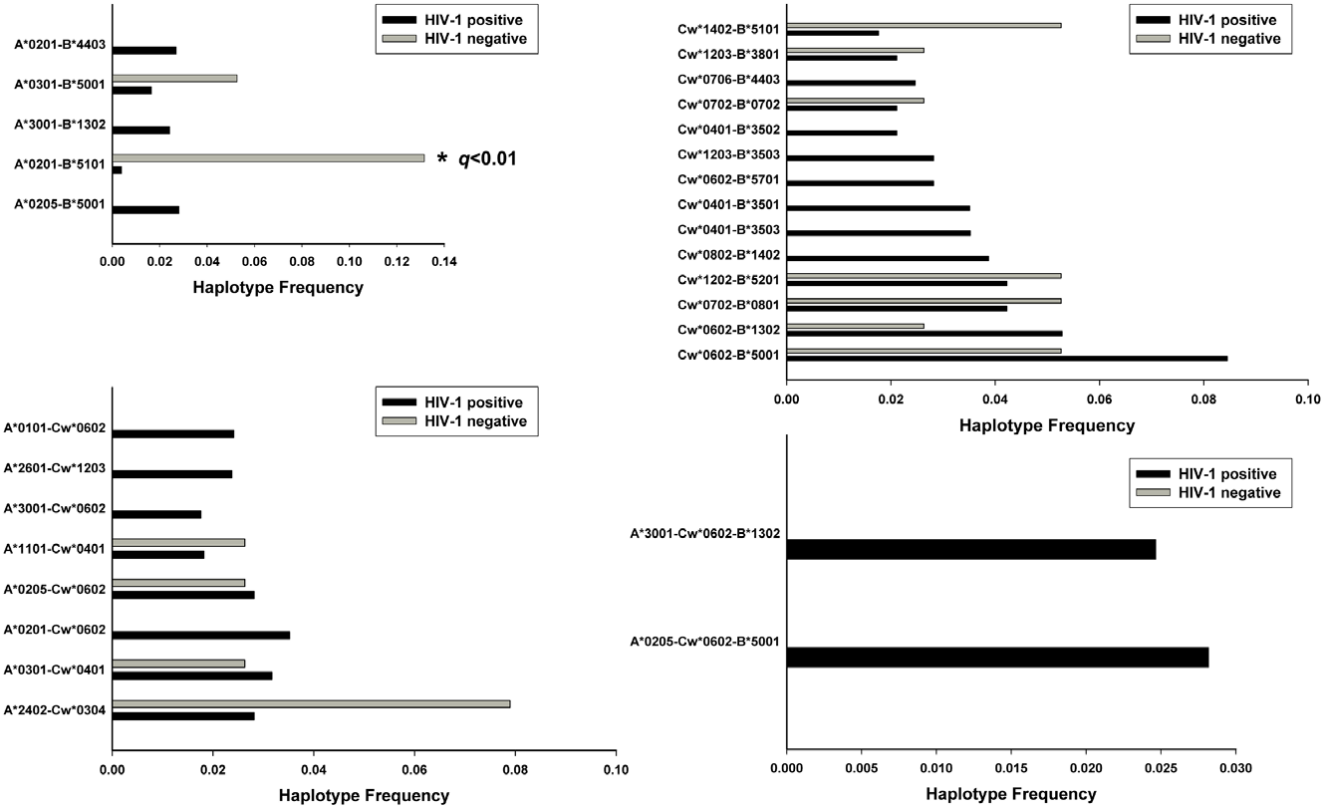
Common HLA class I inferred haplotype frequencies comparison in Uyghur HIV-1 positive and negative cohorts. Only haplotypes with estimated frequencies ≥0.02 are shown. The *q* values refer to comparisons between HIV-1 positive and HIV-1 negative groups.

**Figure 4:**
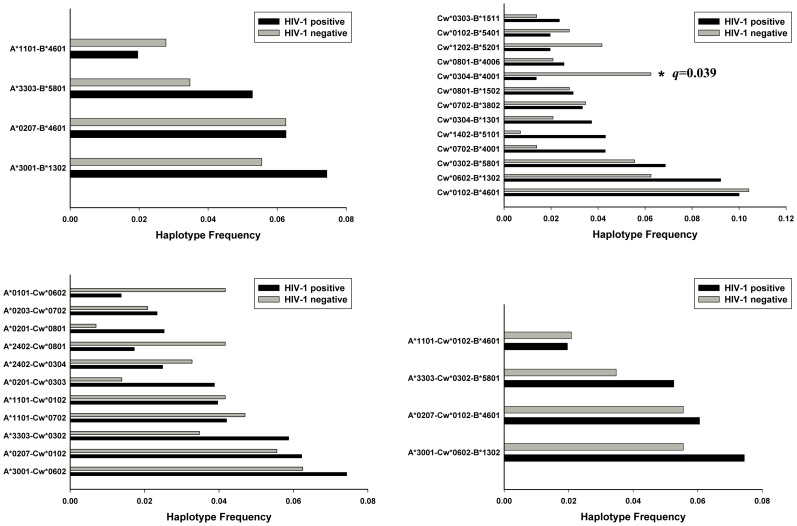
Common HLA class I inferred haplotype frequencies comparison in Han HIV-1 positive and negative cohorts. Only haplotypes with estimated frequencies ≥0.02 are shown. The *q* values refer to comparisons between HIV-1 positive and HIV-1 negative groups.

We compared 2-locus and 3-locus inferred haplotype frequencies in the HIV-1 positive groups and HIV-1 negative groups of our cohorts. The EM algorithm predicted HLA class I haplotypes with an estimated frequency higher than or equal to 0.02 in our Uyghur cohort are presented in Figure 3. In the inferred HLA-A-B haplotypes, the estimated frequency of A*0201-B*5101 (OR = 0.02, 95% CI: 0.003 to 0.21; *p*<0.01, *q*<0.01) was significantly higher in the HIV-1 negative group than in HIV-1 positive group. In our Han cohort, Cw*0304-B*4001 (OR = 0.21, 95% CI: 0.08 to 0.57; *p* = 0.003, q = 0.039) had a significantly higher estimated frequency in the HIV-negative group (Figure 4). We also compared estimated 2-locus and 3-locus haplotypes with frequencies greater than or equal to 0.01 in HIV-1 positive and HIV-1 negative groups (Tables S3 and S4). No statistically significant differences (with a q value lower than 0.2) were observed in these inferred haplotypes in the Uyghur or Han cohort.

### Analysis of phylogenetic tree

**Figure 5:**
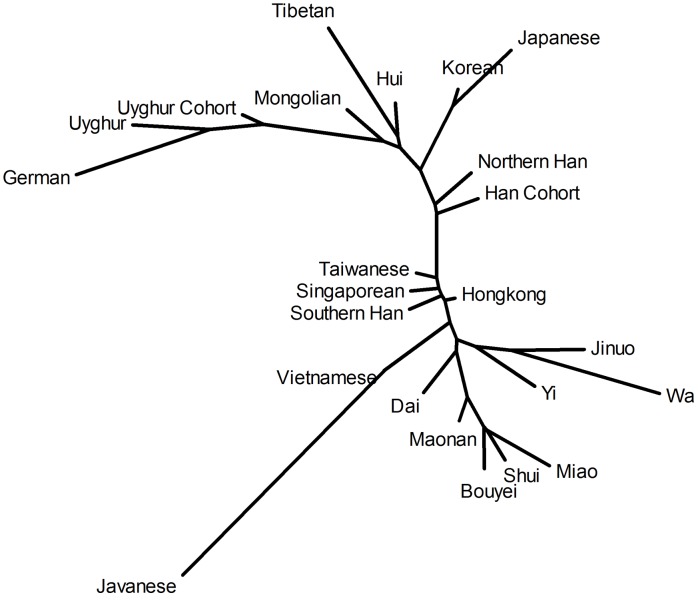
Phylogenetic tree constructed by the neighbor-joining method based on allele frequencies of HLA-A and HLA-B loci shows the relationships of our cohorts with other populations. Uyghur Cohort included our Uyghur HIV-1 positive and negative groups. Han Cohort included our Han HIV-1 positive and negative groups.

A neighbor-joining tree was constructed based on allele frequencies of HLA-A and HLA-B loci (Figure 5). Other Chinese populations included in the phylogenetic tree were Uyghur [19], Tibetan [14], Mongolian and Hui [16], Jinuo and Wa [22], Miao, Bouyie and Shui [23], Maonan [24], Yi [25], Dai [26], Taiwanese [18], Hong Kong and Singapore Chinese [13], Han from southern China living in Hawaii [17], and Han from northern China [15]. Foreign populations included the Kinh population in Vietnam [27], Javanese from Indonesia [28], Korean [29], Japanese [30], and German [31]. Two main clusters were obtained: northern Chinese populations and southern Chinese populations. The northern Chinese cluster included our Uyghur and Han cohorts, Uyghur, northern Han, and northern Chinese minority populations. The German population was the most distant of these. The southern Chinese cluster included the Han populations living in southern China and Southeast Asia, minority populations living in southern China, and the Vietnamese population. The Javanese population was the most distant from these.

## Discussion

Immunogenetic determinants of host susceptibility and resistance to HIV-1 infection have been an area of intense investigation. Increasingly, studies have reported associations between HIV-1 acquisition and low resolution HLA genotypes [20], [21], [32], [33], [34], [35]. However, the 2-digit level data appears insufficient to provide a complete understanding of the role of HLA genotypes. [36]. Four-digit HLA typing may provide more insight into associations between HLA class I profiles and HIV-1 susceptibility and/or disease progression [37]. Few investigations have dealt with the association of HIV-1 susceptibility and 4-digit HLA class I genotypes in Chinese populations.

In this study, we investigated high resolution HLA class I allele distribution and EM predicted haplotype frequencies in Chinese Uyghur and Chinese Han populations. Uyghur blood samples were collected from the Xinjiang Uyghur Autonomous Region, China. Han blood samples were collected from the Sichuan, Henan and Shanxi provinces. Xinjiang, Sichuan, and Henan are among the five provinces in China with the highest reported cases of HIV [38]. Our cohort included members of a large population from a broad region of China, however, analysis of the association of genetic factors with HIV susceptibility was supplemented with previously conducted research on single ethnic groups from specific regions within China [20], [21], [39].

In view of the accumulated evidence suggesting that HLA alleles influence both HIV-1 susceptibility and AIDS progression, the allele frequencies and inferred haplotype frequencies were compared between HIV-1 positive and HIV-1 negative groups within each cohort. In the Han cohort, the frequency of HLA-B*5201 was significantly higher in the HIV-1 negative group. In a study of haemophiliacs in Italy using 2-digit level HLA typing, HLA-B52 was found to be negatively associated with the risk of HIV seroconversion [40]. Epitopes restricted by HLA-B52 are poorly defined. Only one epitope (RMYSPTSI, p24 143–150) has been identified in the HIV Molecular Immunology Database as being restricted by HLA-B*5201 (http://www.hiv.lanl.gov/content/immunology). HLA-Cw18 presents a similar epitope (VRMYSPVSI, p24 142–150). HLA-Cw*18 was found to be protective against HIV in Zambia [41] and Tanzania [37]. Further study of HLA-B*5201 may be relevant to populations beyond China.

In our Uyghur cohort, the frequency of HLA-B*5101 was significantly higher in the HIV-1 negative group. A novel HIV-1 vaccine candidate designed for B*5101 positive individuals protected mice against chimaeric HIV-1 virus [43]. This supports the idea that B*5101 confers some protection from HIV-1 infection. It has also been reported that HLA-B*27, B*57 and B*51 are associated with delayed onset of AIDS [42]. However, the strong association between B*51 and slow disease progression may decline with the adaptation of HIV-1 to HLA induced immune responses [44]. While there may be different mechanisms that result in an allele being associated with protection or slowed disease progression, it is possible that B*5101 is associated with both.

Based on our analysis of published data on the Chinese population [13], [14], [15], [16], [17], [18], [19], [22], [23], [24], [25], [26], allele frequencies of HLA-B*5101 (averaged to 0.044 in northern Chinese vs. 0.038 in southern Chinese) and HLA-B*5201 (averaged to 0.030 in northern Chinese vs. 0.043 in southern Chinese) are not much different in northern and southern Chinese. However, HLA-A*0301, which appeared to be a protective factor in our Han cohort, is a relatively more frequent allele in northern Chinese than in southern Chinese (averaged to 0.042 in northern Chinese vs. 0.005 in southern Chinese). The varied distribution of these alleles in the Chinese population and their associations with HIV-1 susceptibility should be taken into consideration in HIV vaccine development and clinical trial design in China.

HLA phenotype can be grouped as Bw4 serotype and Bw6 serotype according to amino acid residues at positions 77–83 in the α1 domain. In a study with subjects who were mainly Caucasians, infected men carrying the Bw4 allele had lower transmission rates to their female sex partners [45]. The Bw4 homozygosity, Bw6 homozygosity and Bw4/Bw6 heterozygosity did not differ significantly between the HIV-1 positive and negative groups in our cohort. In another study in a Chinese population in Chongqing, Bw4/Bw6 serotype frequencies were not significantly different when HIV-1 positive and negative groups were compared [21]. It is possible that in Chinese populations, bw4/bw6 heterzoygosity or homozygosity may be more associated with AIDS progression [46] or HIV-1 transmission than with susceptibility to infection.

Our data further indicated that 81 Han and 32 Uyghur individuals were homozygous on at least one locus at the 4-digit level. Within this homozygous group, 21 subjects were homozygous on two loci, and 10 were homozygous on all of the three HLA class I loci at the 4-digit level. Homogeneity on HLA class I was not identified to be related to susceptibility to HIV-1 in our cohort.

Considering the significant increase in the frequency of allele HLA-B*5101 in the Uyghur HIV-1 negative group, it is not surprising that there was a statistically significant increase in haplotype A*0201-B*5101 in the Uyghur HIV-1 negative group. Alleles classified in the same supertype can recognize and present epitopes with similar residue hydrophobicity in specific positions. When comparing frequencies of all ten identified supertypes [47], only supertype B62s was found to have a significantly higher frequency (OR = 0.54, 95% CI: 0.36 to 0.81; *p* = 0.003, *q* = 0.012) in the Han HIV-1 negative group than in the Han HIV-1 positive group.

In the neighbor-joining (NJ) phylogenic tree, our Han cohort was grouped in the northern Chinese cluster. Though it had the lowest estimated genetic distance to another northern Han population from Hebei province, the inclusion of subjects from Sichuan may have shifted our cohort slightly closer to Southern Han. Our Uyghur cohort, also in the northern Chinese cluster, included subjects distributed throughout the Xinjiang region. It had the lowest estimated genetic distance to another Uyghur population from Yining, followed by German and Mongolian populations. Previous studies showed that the Uyghur population was a hybrid of 60% European ancestry and 40% East Asian ancestry [48]. The proximity of the Uyghur to the German and Mongolian populations is consistent with these studies and the migration history of the Uyghur population.

Little is known about the mechanism of HLA protection from HIV-1 infection, and it is important to note that association is not equal to a causal relationship. We used statistical analysis to infer associations between alleles and HIV-1 infection or resistance, and this carries a risk of error. Our results are potentially confounded by factors including ethnic population subsets, uneven allele distribution, and proportionally smaller HIV negative cohorts. While efforts were made to select appropriate controls, they may have had a coincidentally lower exposure to HIV. These factors may have minimized some allele associations. Due to the cross-sectional nature of this study, allele associations with protection from HIV-1 infection cannot be distinguished from associations with a delay in seroconversion, and survivorship bias is a risk. We believe the associations presented here are important to consider in our further study, design, and development of HIV vaccine candidates for the Chinese population.

## Materials and Methods

### Ethics statement

The ethnicity of the subjects' parents was identified using questionnaire surveys. All participants provided written informed consent for participation in the study. Subjects enrollment and sampling were approved by the Institutional Review Board (IRB) of the National Center for AIDS and Sexually Transmitted Disease Control and Prevention (NCAIDS) or the IRB of Tangdu Hospital as appropriate. The investigation was conducted in accordance with humane and ethical research principles of Nankai University, China.

### Population

A total of 488 Chinese adults enrolled for this study at NCAIDS in Beijing, and at the Tangdu Hospital affiliated with the Fourth Military Medical University in Xi'an, China. Subjects were confirmed unrelated by collection and comparison of grandparent names. Three hundred and twenty-seven Han subjects were collected from Henan, Shanxi and Sichuan provinces, including 255 HIV-1 seropositive individuals and 72 HIV-1 seronegative individuals. One hundred and sixty-one Uyghur subjects were selected from Xinjiang Uyghur Autonomous Region, including 142 HIV-1 seropositive individuals and 19 HIV-1 seronegative individuals. The subjects ranged from 18 to 65 years of age. To minimize the effects of non-HLA genetic diversity and differences in the risk of exposure, cohorts from each region or province included HIV-1 seropositive and seronegative subjects.

### HLA class I typing

Genomic DNA was extracted from 0.5 ml frozen whole blood using a Qiagen FlexiGene DNA Kit (QIAGEN China Co., Ltd, Shanghai), according to the manufacturer's protocol. HLA-A, HLA-B, and HLA-Cw allele level typing was performed by directly sequencing exon 2 and exon 3 amplification products of HLA loci. Most of the ambiguous allele combinations could be resolved by amplifying and sequencing exon 4 of the HLA-A and HLA-B loci. The HLA specific primers were a gift from Dr. W. H. Hildebrand at the University of Oklahoma Health Sciences Center. The sequence based typing was accomplished with Assign version 3.5 (Conexio Genomics, Applecross, Western Australia, Australia). Class I Direct to High Res SSP UniTray (Invitrogen, USA) was used to resolve the remaining ambiguities.

### Statistical analysis

The HLA class I allele frequencies were calculated by direct counting of the sequencing based typing results. ARLEQUIN software v3.11 [49] was used to estimate the haplotypes (2-locus and 3-locus) using the maximum-likelihood method, with the iterative EM algorithm. POPTREE2 was used to calculate genetic distance between different populations. A neighbor-joining phylogenetic tree based on allele frequencies of HLA-A and HLA-B loci was constructed with POPTREE2 [50].

The comparison of Chinese Han HLA class I allele frequencies between HIV-1 seropositive and seronegative groups was performed using the Cochran-Mantel-Haenszel Chi-squared test with PASW statistics 18.0. Since our Chinese Han subjects were selected from three provinces in China, the location was selected as a layer variable. The comparison of Chinese Uyghur HLA class I allele frequencies between HIV-1 seropositive and seronegative groups was performed using Chi-squared test. The comparisons of Chinese Han and Uyghur HLA class I haplotype frequencies between HIV-1 seropositive and seronegative groups were performed using Chi-squared test. Fisher exact test (two tailed) or Yates's continuity correction was applied when necessary. The strength of an association was indicated by an odds ratio (OR) with a 95% confidence interval (CI) calculated with PASW software. In consideration of the multiple comparisons performed, *p* values were used to calculate *q* values to control the false discovery rate with the false discovery rate (FDR) method described by Benjamini and Hochberg. Comparisons with *q* values less than 0.2 were accepted as statistically significant associations.

## Supporting Information

Table S1 Distribution of common HLA-A*, Cw* and B* alleles among Chinese Uyghur HIV-1 positive and negative subjects.
(DOCX)

Table S2 Distribution of common HLA-A*, Cw* and B* alleles among Chinese Han HIV-1 positive and negative subjects.
(DOCX)

Table S3 **Distribution of common HLA class I haplotypes among Chinese Uyghur HIV-1 positive and negative subjects.**
(DOCX)

Table S4 **Distribution of common HLA class I haplotypes among Chinese Han HIV-1 positive and negative subjects.**
(DOCX)
