# Supplementary material for: High Resolution Human Leukocyte Antigen Class I Allele Frequencies and HIV-1 Infection Associations in Chinese Han and Uyghur Cohorts
Source: PLoS One. 2012 Dec 12;7(12):e50656. doi: 10.1371/journal.pone.0050656 (PMC3520934; doi:10.1371/journal.pone.0050656)
Supplement: Table S1 — Distribution of common HLA-A*, Cw* and B* alleles among Chinese Uyghur HIV-1 positive and negative subjects. (DOCX) [file pone.0050656.s001.docx]

**Table S1. Distribution of common HLA-A*, Cw* and B* alleles among Chinese Uyghur HIV-1 positive and negative subjects.**

| **HLA alleles** | **HIV-1 positive frequency** | **HIV-1 negative frequency** | ***p* value** | ***q* value** | **OR** | **95% CI** |
| --- | --- | --- | --- | --- | --- | --- |
| A* 0201 | 0.120 | 0.237 | 0.071 |  | 0.44 | 0.19-1.00 |
| A* 2402 | 0.102 | 0.211 | 0.060 |  | 0.43 | 0.18-1.02 |
| A* 1101 | 0.099 | 0.105 | 0.779 |  | 0.93 | 0.31-2.81 |
| A* 0301 | 0.095 | 0.105 | 0.773 |  | 0.89 | 0.29-2.71 |
| A* 0101 | 0.102 | 0.026 | 0.229 |  | 4.21 | 0.56-31.82 |
| A* 2601 | 0.053 | 0.026 | 0.704 |  | 2.06 | 0.27-16.08 |
| A* 3303 | 0.035 | 0.026 | 1.000 |  | 1.35 | 0.17-10.85 |
| A* 0205 | 0.032 | 0.026 | 1.000 |  | 1.21 | 0.15-9.83 |
| A* 0207 | 0.032 | 0.026 | 1.000 |  | 1.21 | 0.15-9.83 |
| A* 3001 | 0.035 | 0.000 | 0.498 |  | 0.98 | 0.12-8.20 |
| A* 0206 | 0.021 | 0.053 | 0.241 |  | 0.39 | 0.08-2.00 |
| A* 6801 | 0.025 | 0.026 | 1.000 |  | 0.94 | 0.11-7.82 |
| A* 2301 | 0.025 | 0.000 | 1.000 |  | 1.13 | 0.14-9.25 |
| A* 0211 | 0.021 | 0.000 | 1.000 |  | 0.98 | 0.12-8.20 |
| A* 0302 | 0.021 | 0.000 | 1.000 |  | 0.98 | 0.12-8.20 |
| A* 3101 | 0.014 | 0.053 | 0.150 |  | 0.26 | 0.05-1.45 |
| A* 3201 | 0.018 | 0.000 | 1.000 |  | 0.84 | 0.10-7.15 |
| A* 3301 | 0.021 | 0.000 | 1.000 |  | 0.98 | 0.12-8.20 |
| A* 6802 | 0.018 | 0.000 | 1.000 |  | 0.84 | 0.10-7.15 |
| A* 1102 | 0.014 | 0.000 | 1.000 |  | 0.70 | 0.08-6.12 |
| A* 3004 | 0.014 | 0.000 | 1.000 |  | 0.70 | 0.08-6.12 |
| Cw* 0602 | 0.190 | 0.079 | 0.092 |  | 2.74 | 0.81-9.24 |
| Cw* 0401 | 0.120 | 0.105 | 1.000 |  | 1.16 | 0.39-3.46 |
| Cw* 0702 | 0.102 | 0.079 | 1.000 |  | 1.33 | 0.38-4.59 |
| Cw* 1203 | 0.081 | 0.053 | 0.751 |  | 1.59 | 0.36-7.01 |
| Cw* 0304 | 0.060 | 0.105 | 0.291 |  | 0.54 | 0.17-1.7 |
| Cw* 1202 | 0.053 | 0.079 | 0.456 |  | 0.65 | 0.18-2.36 |
| Cw* 0102 | 0.056 | 0.026 | 0.704 |  | 2.21 | 0.29-17.15 |
| Cw* 0701 | 0.039 | 0.079 | 0.221 |  | 0.47 | 0.13-1.77 |
| Cw* 1502 | 0.028 | 0.105 | 0.041 |  | 0.25 | 0.07-0.86 |
| Cw* 0802 | 0.039 | 0.000 | 0.374 |  | 1.57 | 0.20-12.46 |
| Cw* 0706 | 0.032 | 0.000 | 0.606 |  | 1.27 | 0.16-10.31 |
| Cw* 0801 | 0.028 | 0.000 | 0.606 |  | 1.13 | 0.14-9.25 |
| Cw* 0303 | 0.021 | 0.026 | 0.588 |  | 0.80 | .09-6.82 |
| Cw* 0727 | 0.025 | 0.000 | 1.000 |  | 1.13 | 0.14-9.25 |
| Cw* 1402 | 0.018 | 0.053 | 0.195 |  | 0.32 | 0.06-1.72 |
| Cw* 0302 | 0.021 | 0.000 | 1.000 |  | 0.98 | 0.12-8.20 |
| Cw* 0501 | 0.018 | 0.026 | 0.532 |  | 0.66 | 0.08-5.83 |
| Cw* 1602 | 0.018 | 0.026 | 0.532 |  | 0.66 | 0.08-5.83 |
| Cw* 1701 | 0.018 | 0.026 | 0.532 |  | 0.66 | 0.08-5.83 |
| B* 5001 | 0.092 | 0.079 | 1.000 |  | 1.18 | 0.34-4.09 |
| B* 3503 | 0.063 | 0.000 | 0.145 |  | 2.63 | 0.34-20.25 |
| **B* 5101** | **0.039** | **0.184** | **0.002** | **0.056** | **0.18** | **0.06-0.49** |
| B* 0801 | 0.053 | 0.053 | 1.000 |  | 1.00 | 0.22-4.57 |
| B* 1302 | 0.053 | 0.026 | 0.704 |  | 2.06 | 0.27-16.08 |
| B* 5201 | 0.046 | 0.053 | 0.693 |  | 0.86 | 0.19-3.98 |
| B* 4403 | 0.042 | 0.053 | 0.675 |  | 0.79 | 0.17-3.69 |
| B* 1402 | 0.042 | 0.000 | 0.373 |  | 1.86 | 0.24-14.65 |
| B* 3501 | 0.039 | 0.026 | 1.000 |  | 1.49 | 0.19-11.88 |
| B* 4001 | 0.032 | 0.026 | 1.000 |  | 1.21 | 0.15-9.83 |
| B* 1801 | 0.028 | 0.026 | 1.000 |  | 1.07 | 0.13-8.82 |
| B* 4006 | 0.025 | 0.053 | 0.288 |  | 0.46 | 0.09-2.27 |
| B* 4101 | 0.025 | 0.053 | 0.288 |  | 0.46 | 0.09-2.27 |
| B* 0702 | 0.025 | 0.026 | 1.000 |  | 0.94 | 0.11-7.82 |
| B* 1501 | 0.025 | 0.026 | 1.000 |  | 0.94 | 0.11-7.82 |
| B* 3801 | 0.025 | 0.026 | 1.000 |  | 0.94 | 0.11-7.82 |
| B* 4601 | 0.025 | 0.026 | 1.000 |  | 0.94 | 0.11-7.82 |
| B* 5701 | 0.028 | 0.000 | 0.603 |  | 1.13 | 0.14-9.25 |
| B* 3502 | 0.021 | 0.026 | 0.588 |  | 0.80 | 0.09-6.82 |
| B* 4402 | 0.018 | 0.053 | 0.195 |  | 0.32 | 0.06-1.72 |
| B* 3701 | 0.021 | 0.000 | 1.000 |  | 0.98 | 0.12-8.20 |
| B* 3802 | 0.021 | 0.000 | 1.000 |  | 0.98 | 0.12-8.20 |
| B* 3901 | 0.018 | 0.026 | 0.532 |  | 0.66 | 0.08-5.83 |
| B* 5501 | 0.018 | 0.026 | 0.532 |  | 0.66 | 0.08-5.83 |
| B* 5801 | 0.021 | 0.000 | 1.000 |  | 0.98 | 0.12-8.20 |
| B* 4801 | 0.011 | 0.053 | 0.108 |  | 0.19 | 0.03-1.19 |
| B* 1301 | 0.014 | 0.000 | 1.000 |  | 0.70 | 0.08-6.11 |
| B* 4901 | 0.011 | 0.026 | 0.396 |  | 0.40 | 0.04-3.90 |

HIV-1 positive 2n = 284. HIV-1 negative 2n = 38. Only alleles with frequencies ≥ 0.01 are shown. The *p* values and *q* values refer to comparisons between HIV-1 positive and HIV-1 negative groups.
