# Supplementary material for: High Resolution Human Leukocyte Antigen Class I Allele Frequencies and HIV-1 Infection Associations in Chinese Han and Uyghur Cohorts
Source: PLoS One. 2012 Dec 12;7(12):e50656. doi: 10.1371/journal.pone.0050656 (PMC3520934; doi:10.1371/journal.pone.0050656)
Supplement: Table S2 — Distribution of common HLA-A*, Cw* and B* alleles among Chinese Han HIV-1 positive and negative subjects. (DOCX) [file pone.0050656.s002.docx]

**Table S2. Distribution of common HLA-A*, Cw* and B* alleles among Chinese Han HIV-1 positive and negative subjects.**

| **HLA alleles** | **HIV-1 positive frequency** | **HIV-1 negative frequency** | ***p* value** | ***q* value** | **OR** | **95% CI** |
| --- | --- | --- | --- | --- | --- | --- |
| A*1101 | 0.157 | 0.160 | 0.464 |  | 1.21 | 0.72-2.02 |
| A*2402 | 0.135 | 0.139 | 0.951 |  | 1.01 | 0.58-1.78 |
| A*0201 | 0.131 | 0.125 | 0.789 |  | 0.92 | 0.52-1.65 |
| A*3303 | 0.092 | 0.063 | 0.267 |  | 1.53 | 0.72-3.26 |
| A*3001 | 0.084 | 0.076 | 0.889 |  | 1.05 | 0.52-2.13 |
| A*0207 | 0.075 | 0.069 | 0.770 |  | 1.12 | 0.53-2.38 |
| A*0206 | 0.061 | 0.049 | 0.542 |  | 1.30 | 0.55-3.08 |
| A*0203 | 0.035 | 0.049 | 0.581 |  | 0.76 | 0.29-1.98 |
| A*3101 | 0.043 | 0.021 | 0.136 |  | 2.47 | 0.72-8.47 |
| **A*0301** | **0.022** | **0.063** | **0.002** | **0.030** | **0.25** | **0.09-0.64** |
| A*0101 | 0.022 | 0.056 | 0.044 |  | 0.39 | 0.17-1.02 |
| A*2601 | 0.024 | 0.028 | 0.855 |  | 1.12 | 0.34-3.70 |
| A*2901 | 0.020 | 0.007 | 0.393 |  | 2.38 | 0.30-18.84 |
| A*1102 | 0.016 | 0.014 | 0.829 |  | 1.22 | 0.22-6.73 |
| A*3201 | 0.014 | 0.014 | 0.480 |  | 0.54 | 0.10-2.94 |
| Cw*0102 | 0.165 | 0.188 | 0.820 |  | 0.94 | 0.57-1.55 |
| Cw*0702 | 0.135 | 0.132 | 0.341 |  | 1.34 | 0.75-2.39 |
| Cw*0602 | 0.122 | 0.111 | 0.880 |  | 0.96 | 0.53-1.73 |
| Cw*0801 | 0.088 | 0.083 | 0.971 |  | 0.99 | 0.51-1.94 |
| Cw*0304 | 0.067 | 0.104 | 0.133 |  | 0.60 | 0.31-1.17 |
| Cw*0302 | 0.076 | 0.056 | 0.288 |  | 1.56 | 0.69-3.51 |
| Cw*0303 | 0.073 | 0.049 | 0.419 |  | 1.41 | 0.61-3.27 |
| Cw*0401 | 0.053 | 0.069 | 0.324 |  | 0.67 | 0.30-1.48 |
| Cw*1402 | 0.051 | 0.007 | 0.072 |  | 2.96 | 0.86-10.22 |
| Cw*1502 | 0.041 | 0.021 | 0.340 |  | 1.88 | 0.52-6.85 |
| Cw*1202 | 0.024 | 0.042 | 0.028 |  | 0.31 | 0.11-0.91 |
| Cw*1505 | 0.016 | 0.014 | 0.840 |  | 0.85 | 0.17-4.16 |
| Cw*0202 | 0.014 | 0.007 | 0.251 |  | 3.39 | 0.39-29.65 |
| Cw*0803 | 0.012 | 0.007 | 0.778 |  | 1.37 | 0.16-11.84 |
| Cw*1403 | 0.010 | 0.014 | 0.283 |  | 0.75 | 0.19-2.95 |
| B*4601 | 0.104 | 0.118 | 0.826 |  | 0.93 | 0.50-1.73 |
| B*1302 | 0.092 | 0.063 | 0.395 |  | 1.37 | 0.65-2.88 |
| B*4001 | 0.065 | 0.083 | 0.626 |  | 0.84 | 0.41-1.71 |
| B*5801 | 0.069 | 0.063 | 0.520 |  | 1.30 | 0.59-2.86 |
| B*5101 | 0.065 | 0.021 | 0.053 |  | 3.06 | 0.92-10.20 |
| B*3501 | 0.045 | 0.035 | 0.639 |  | 0.78 | 0.28-2.20 |
| **B*5201** | **0.027** | **0.083** | **0.001** | **0.026** | **0.24** | **0.10-0.57** |
| B*1301 | 0.043 | 0.021 | 0.229 |  | 2.18 | 0.61-7.76 |
| B*1501 | 0.031 | 0.063 | 0.038 |  | 0.42 | 0.18-0.99 |
| B*3802 | 0.039 | 0.035 | 0.494 |  | 1.47 | 0.51-4.28 |
| B*4006 | 0.033 | 0.021 | 0.827 |  | 1.14 | 0.33-3.96 |
| B*4801 | 0.033 | 0.021 | 0.526 |  | 1.47 | 0.43-5.05 |
| B*1502 | 0.029 | 0.028 | 0.822 |  | 1.13 | 0.37-3.42 |
| B*4403 | 0.022 | 0.042 | 0.130 |  | 0.45 | 0.16-1.28 |
| B*1511 | 0.024 | 0.021 | 0.773 |  | 1.22 | 0.32-4.70 |
| B*4002 | 0.024 | 0.021 | 0.715 |  | 1.28 | 0.34-4.83 |
| B*5401 | 0.022 | 0.028 | 0.772 |  | 0.80 | 0.24-2.67 |
| B*0702 | 0.022 | 0.007 | 0.334 |  | 2.66 | 0.33-21.18 |
| B*0705 | 0.016 | 0.014 | 0.840 |  | 0.85 | 0.17-4.16 |
| B*3901 | 0.012 | 0.021 | 0.486 |  | 0.59 | 0.13-2.65 |
| B*5502 | 0.014 | 0.014 | 0.957 |  | 0.96 | 0.19-4.90 |
| B*1525 | 0.010 | 0.021 | 0.608 |  | 0.66 | 0.14-3.18 |
| B*2705 | 0.014 | 0.007 | 0.251 |  | 3.39 | 0.39-29.65 |
| B*3701 | 0.010 | 0.021 | 0.262 |  | 0.45 | 0.10-1.94 |
| B*0801 | 0.012 | 0.007 | 0.404 |  | 2.55 | 0.28-23.50 |
| B*5701 | 0.008 | 0.021 | 0.110 |  | 0.29 | 0.06-1.39 |

HIV-1 positive 2n = 510. HIV-1 negative 2n = 144. Only alleles with frequencies ≥ 0.01 are shown. The *p* values and *q* values refer to comparisons between HIV-1 positive and HIV-1 negative groups.
