# Supplementary material for: High Resolution Human Leukocyte Antigen Class I Allele Frequencies and HIV-1 Infection Associations in Chinese Han and Uyghur Cohorts
Source: PLoS One. 2012 Dec 12;7(12):e50656. doi: 10.1371/journal.pone.0050656 (PMC3520934; doi:10.1371/journal.pone.0050656)
Supplement: Table S3 — Distribution of common HLA class I haplotypes among Chinese Uyghur HIV-1 positive and negative subjects. (DOCX) [file pone.0050656.s003.docx]

**Table S3. Distribution of common HLA class I haplotypes among Chinese Uyghur HIV-1 positive and negative subjects.**

| **Haplotypes** | **HIV-1 positive frequency** | **HIV-1 negative frequency** | ***p* value** | ***q* value** | **OR** | **95% CI** |
| --- | --- | --- | --- | --- | --- | --- |
| A*0205-B*5001 | 0.028 | 0.000 | 0.603 |  | 1.13 | 0.14-9.25 |
| **A*0201-B*5101** | **0.004** | **0.132** | **<0.000** | **<0.000** | **0.02** | **0.003-0.21** |
| A*3001-B*1302 | 0.024 | 0.000 | 1.000 |  | 0.98 | 0.12-8.20 |
| A*0301-B*5001 | 0.017 | 0.053 | 0.195 |  | 0.32 | 0.06-1.72 |
| A*0201-B*4403 | 0.027 | 0.000 | 0.603 |  | 1.13 | 0.14-9.25 |
| Cw*0602-B*5001 | 0.085 | 0.053 | 0.996 |  | 1.00 | 0.22-4.57 |
| Cw*0602-B*1302 | 0.053 | 0.026 | 1.000 |  | 1.63 | 0.21-12.92 |
| Cw*0702-B*0801 | 0.042 | 0.053 | 0.675 |  | 0.79 | 0.17-3.69 |
| Cw*1202-B*5201 | 0.042 | 0.053 | 0.657 |  | 0.73 | 0.15-3.40 |
| Cw*0802-B*1402 | 0.039 | 0.000 | 0.614 |  | 1.42 | 0.18-11.38 |
| Cw*0401-B*3503 | 0.035 | 0.000 | 0.614 |  | 1.42 | 0.18-11.38 |
| Cw*0401-B*3501 | 0.035 | 0.000 | 0.603 |  | 1.13 | 0.14-9.25 |
| Cw*0602-B*5701 | 0.028 | 0.000 | 0.603 |  | 1.13 | 0.14-9.25 |
| Cw*1203-B*3503 | 0.028 | 0.000 | 1.000 |  | 0.98 | 0.12-8.20 |
| Cw*0401-B*3502 | 0.021 | 0.000 | 1.000 |  | 0.98 | 0.12-8.20 |
| Cw*0702-B*0702 | 0.021 | 0.026 | 1.000 |  | 0.94 | 0.11-7.82 |
| Cw*0706-B*4403 | 0.025 | 0.000 | 1.000 |  | 0.98 | 0.12-8.20 |
| Cw*1203-B*3801 | 0.021 | 0.026 | 0.532 |  | 0.66 | 0.08-5.83 |
| Cw*1402-B*5101 | 0.018 | 0.053 | 0.195 |  | 0.32 | 0.06-1.72 |
| A*2402-Cw*0304 | 0.028 | 0.079 | 0.128 |  | 0.34 | 0.09-1.33 |
| A*0301-Cw*0401 | 0.032 | 0.026 | 1.000 |  | 1.21 | 0.15-9.83 |
| A*0201-Cw*0602 | 0.035 | 0.000 | 0.614 |  | 1.42 | 0.18-11.38 |
| A*0205-Cw*0602 | 0.028 | 0.026 | 1.000 |  | 1.07 | 0.13-8.82 |
| A*1101-Cw*0401 | 0.018 | 0.026 | 0.532 |  | 0.66 | 0.08-5.83 |
| A*3001-Cw*0602 | 0.018 | 0.000 | 1.000 |  | 0.84 | 0.10-7.15 |
| A*2601-Cw*1203 | 0.024 | 0.000 | 1.000 |  | 0.98 | 0.12-8.20 |
| A*0101-Cw*0602 | 0.024 | 0.000 | 1.000 |  | 0.98 | 0.12-8.20 |
| A*0205-Cw*0602-B*5001 | 0.028 | 0.000 | 0.603 |  | 1.13 | 0.14-9.25 |
| A*3001-Cw*0602-B*1302 | 0.025 | 0.000 | 1.000 |  | 1.13 | 0.14-9.25 |

Only haplotypes with frequencies ≥ 0.02 are shown. The *p* values and *q* values refer to comparisons between HIV-1 positive and HIV-1 negative groups.
