# Supplementary material for: High Resolution Human Leukocyte Antigen Class I Allele Frequencies and HIV-1 Infection Associations in Chinese Han and Uyghur Cohorts
Source: PLoS One. 2012 Dec 12;7(12):e50656. doi: 10.1371/journal.pone.0050656 (PMC3520934; doi:10.1371/journal.pone.0050656)
Supplement: Table S4 — Distribution of common HLA class I haplotypes among Chinese Han HIV-1 positive and negative subjects. (DOCX) [file pone.0050656.s004.docx]

**Table S4. Distribution of common HLA class I haplotypes among Chinese Han HIV-1 positive and negative subjects.**

| **Haplotypes** | **HIV-1 positive frequency** | **HIV-1 negative frequency** | ***p* value** | ***q* value** | **OR** | **95% CI** |
| --- | --- | --- | --- | --- | --- | --- |
| A*3001-B*1302 | 0.074 | 0.056 | 0.432 |  | 1.37 | 0.62-3.00 |
| A*0207-B*4601 | 0.063 | 0.063 | 0.991 |  | 1.00 | 0.47-2.16 |
| A*3303-B*5801 | 0.053 | 0.035 | 0.371 |  | 1.55 | 0.59-4.11 |
| A*1101-B*4601 | 0.020 | 0.028 | 0.552 |  | 0.70 | 0.22-2.27 |
| Cw*0102-B*4601 | 0.100 | 0.104 | 0.883 |  | 0.96 | 0.52-1.76 |
| Cw*0602-B*1302 | 0.092 | 0.063 | 0.261 |  | 1.52 | 0.73-3.19 |
| Cw*0302-B*5801 | 0.069 | 0.056 | 0.576 |  | 1.25 | 0.57-2.76 |
| Cw*0702-B*4001 | 0.043 | 0.014 | 0.099 |  | 3.20 | 0.74-13.78 |
| Cw*1402-B*5101 | 0.043 | 0.007 | 0.037 |  | 6.45 | 0.86-48.24 |
| Cw*0304-B*1301 | 0.037 | 0.021 | 0.439 |  | 1.82 | 0.53-6.24 |
| Cw*0702-B*3802 | 0.033 | 0.035 | 1.000 |  | 0.96 | 0.35-2.64 |
| Cw*0801-B*1502 | 0.029 | 0.028 | 1.000 |  | 1.06 | 0.35-3.25 |
| **Cw*0304-B*4001** | **0.014** | **0.063** | **0.003** | **0.039** | **0.21** | **0.08-0.57** |
| Cw*0801-B*4006 | 0.025 | 0.021 | 1.000 |  | 1.23 | 0.35-4.37 |
| Cw*1202-B*5201 | 0.020 | 0.042 | 0.134 |  | 0.46 | 0.16-1.29 |
| Cw*0102-B*5401 | 0.020 | 0.028 | 0.522 |  | 0.70 | 0.22-2.27 |
| Cw*0303-B*1511 | 0.024 | 0.014 | 0.745 |  | 1.71 | 0.38-7.73 |
| A*3001-Cw*0602 | 0.074 | 0.063 | 0.622 |  | 1.21 | 0.57-2.56 |
| A*0207-Cw*0102 | 0.062 | 0.056 | 0.751 |  | 1.14 | 0.51-2.53 |
| A*3303-Cw*0302 | 0.059 | 0.035 | 0.256 |  | 1.74 | 0.66-4.56 |
| A*1101-Cw*0702 | 0.042 | 0.047 | 0.697 |  | 0.84 | 0.35-2.02 |
| A*1101-Cw*0102 | 0.040 | 0.042 | 0.894 |  | 0.94 | 0.37-2.38 |
| A*0201-Cw*0303 | 0.039 | 0.014 | 0.191 |  | 2.90 | 0.67-12.55 |
| A*2402-Cw*0304 | 0.025 | 0.033 | 0.565 |  | 0.73 | 0.26-2.08 |
| A*2402-Cw*0801 | 0.017 | 0.042 | 0.111 |  | 0.41 | 0.15-1.18 |
| A*0201-Cw*0801 | 0.025 | 0.007 | 0.324 |  | 3.74 | 0.49-28.84 |
| A*0203-Cw*0702 | 0.023 | 0.021 | 1.000 |  | 1.13 | 0.32-4.07 |
| A*0101-Cw*0602 | 0.014 | 0.042 | 0.045 |  | 0.32 | 0.11-0.97 |
| A*3001-Cw*0602-B*1302 | 0.074 | 0.056 | 0.432 |  | 1.37 | 0.62-3.00 |
| A*0207-Cw*0102-B*4601 | 0.061 | 0.056 | 0.815 |  | 1.10 | 0.49-2.45 |
| A*3303-Cw*0302-B*5801 | 0.053 | 0.035 | 0.371 |  | 1.55 | 0.59-4.11 |
| A*1101-Cw*0102-B*4601 | 0.020 | 0.021 | 1.000 |  | 0.94 | 0.26-3.46 |

Only haplotypes with frequencies ≥ 0.02 are shown. The *p* values and *q* values refer to comparisons between HIV-1 positive and HIV-1 negative groups.
